# Innovative approaches to refractive error services: criteria and considerations for success

**Published:** 2022-06-07

**Authors:** Jude Stern

**Affiliations:** 1 Head of Knowledge Management: IAPB, Sydney, Australia.

**Innovative approaches and technology-based solutions have a significant role to play to ensure that equitable and accessible eye health services are available to all.**

**Figure:**
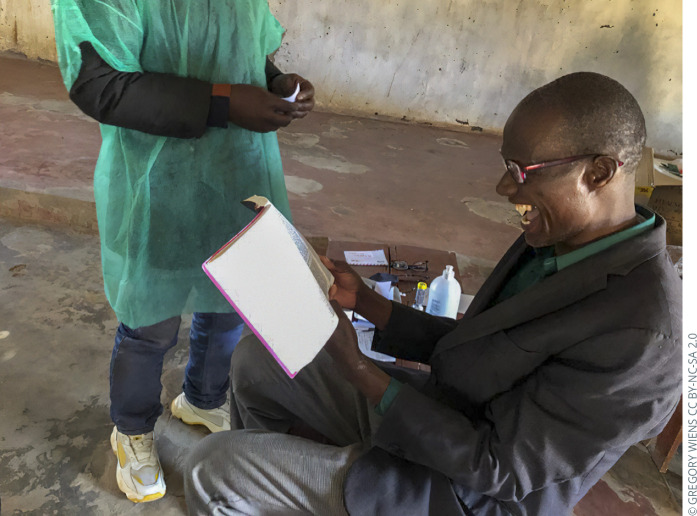
Getting a pair of spectacles can be life-changing. **MALAWI**

The success and sustainability of a new technology depends on how accurate and safe it is, and how well it meets the specific needs of the country or community it is meant to serve.

Technology-based solutions to enable access to refractive error care have been fast tracked during the pandemic, but evidence of their success and sustainability is mixed. To meet the need for guidance in this area, the IAPB Refractive Error Working Group recently published a paper (https://bit.ly/RE-paper) which defines minimum criteria and recommendations for the quality and operating environment of screening, self-refraction, and self-prescribing devices.

The minimum criteria in the paper can be used by the manufacturers and users of refractive error technologies to prepare for the successful introduction and sustained, ongoing use of these technologies to deliver equitable access to refractive error services.

## Efficacy, performance and quality

### Screening devices

The introduction of tablet and smartphone devices has increased access to vision screening, opening the potential for people to self-screen. Screening results can be digitally and automatically added to patients’ digital health record and a positive test can also trigger a referral.

Ensuring the accuracy of these devices, and the competency of the personnel trained to use them, is critical. An excess of false positives can overload referral sites, lead to unnecessary expenditure by patients and reduce confidence in the system, whereas an excess of false negatives means patients will not get the refractive error correction they need.

### Criteria and considerations for introduction

1. Screening devices consistently achieve minimum screening thresholds for detection of vision impairment, validated against clinically trained screeners using traditional manual methods.
2. The devices have been tested and validated in the country of introduction or a similar country context, and the data and results are published in a peer-reviewed journal.
3. Devices are easy for screeners to understand and use.
4. Mechanisms are in place to monitor and assess the effectiveness of the device and its implementation.
5. The device is appropriate for children, to the required standard.

### Self-refraction and prescribing devices

Shortages of trained health workers to carry out refractions have led to innovations in self-refraction by means of various adjustable spectacle designs or smartphones, which can increase access for many communities. Caution should be used with self-refraction devices and adjustable spectacles for children aged 10 years and under, as these have not yet been sufficiently studied to determine their accuracy and efficacy.

### Criteria and considerations for introduction

1. The device has undergone testing that establishes a significant correlation between conventional refraction and the refraction received by the self-refraction testing device, defined as 95% of patients achieving visual acuity of 6/12 and/or N6 or better when using the device.
2. The device has been tested and validated in the country of introduction or in a similar country context, and the data and results are published in a peer-reviewed journal.
3. The organisation implementing the use of the device is competent and active in conveying basic eye health information to the patients and is aware of existing referral pathways.
4. Device maintenance, repair, and replacement options are available locally or regionally.

## Context and operating environment

**Figure 1:**
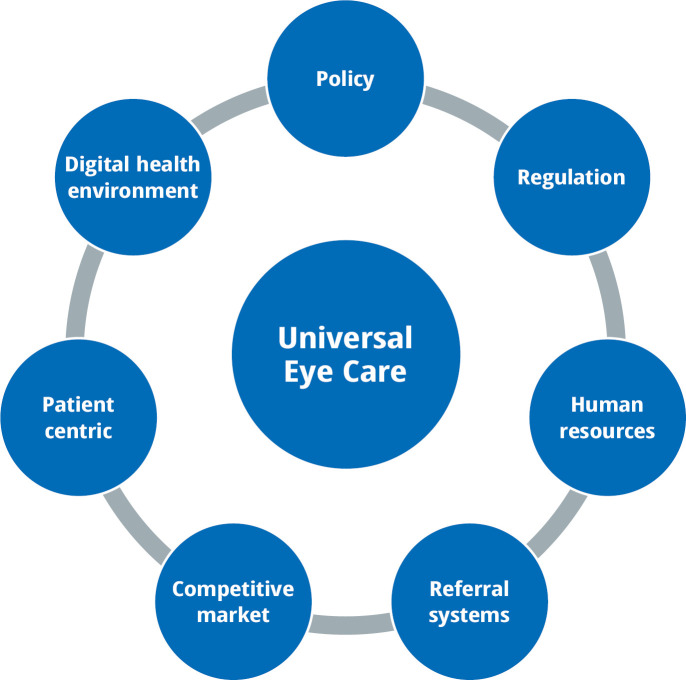
When introducing new technology for refractive error care, carefully consider the broader ecosystem in which it will be deployed.

To set up for success, tailor any new refractive error innovation to the local or national context. Differences related to the digital health environment, policy, regulation, human resources, referral systems, and the competitive market environment (Figure 1) will determine the uptake and use of new technology and therefore its potential for facilitating equitable and permanent access to refractive error services in the community.

Invite local, national, and global stakeholders to help assess the relevance of the new technology and its potential for meeting the needs in that setting, compared to other models. The same stakeholders should also be involved in validating interoperability and integration with existing systems and establishing processes that will ensure that the technology can achieve positive eye health outcomes and benefit the health system in the short and long term.

### Ensuring equity

When introducing new technology, consider from the outset how you will reach the most difficult-to-reach groups; that is, how you will ensure equitable access. It's just as important to continue to measure equity of access by gathering information about groups of concern (e.g., people with disabilities, women, and minority ethnic groups) so that you can monitor the level and type of coverage and put protocols in place to address inequity. Any service involving children must be appropriate for children and have safeguarding protocols in place and activated.

To read more, the IAPB position paper on refractive error technologies (https://bit.ly/RE-paper) further outlines minimum criteria and recommendations for each of these elements of the operating environment and the quality of refractive error technologies.

*This is a summary of a position paper from the Refractive Error Working Group of the International Agency for the Prevention of Blindness (IAPB).*
